# Acute to Chronic Electro-Clinical Manifestations of Neuro-COVID and the Long-Haul Consequences in People With Epilepsy: A Review

**DOI:** 10.7759/cureus.26020

**Published:** 2022-06-16

**Authors:** Anna Milan, Philippe Salles, Carolina Pelayo, Reinaldo Uribe-San-Martin

**Affiliations:** 1 Epilepsy, Liga Chilena Contra La Epilepsia, Santiago, CHL; 2 Movement Disorders, Centro de Trastornos del Movimiento (CETRAM), Santiago, CHL; 3 Neuroimmunology, Pontificia Universidad Católica de Chile, Santiago, CHL; 4 Epilepsy, Pontificia Universidad Catolica de Chile, Santiago, CHL

**Keywords:** people with epilepsy, brain fog, eeg, long-haul covid, encephalitis, covid-19, seizures, epilepsy

## Abstract

Severe acute respiratory syndrome coronavirus 2 (SARS-COV-2) infection can involve the central nervous system (CNS). Acute symptomatic seizures or epileptiform discharges have not been commonly reported in patients with altered mental status related to coronavirus disease 2019 (COVID-19) infection. However, long-term neurological symptoms have been reported after COVID-19 infection (i.e., brain fog, cognitive complaints, and confusion), suggesting chronic encephalopathy. People with epilepsy (PWE) have been specifically affected by the COVID-19 pandemic, with changes in their seizure frequency, quality of life, health care accessibility, and medication interactions. This narrative review highlights possible pathophysiological mechanisms of COVID-19 on the brain, related to short- and long-term epileptiform activity and the impact of this infection on PWE.

## Introduction and background

Coronavirus disease 2019 (COVID-19) has affected more than 508 million people, causing more than 6 million deaths, as reported by World Health Organization (https://covid19.who.int). In addition to respiratory symptoms, this disease compromises the central nervous system (CNS) [1]. Acutely, patients may manifest mild to severe encephalitis, with unspecific symptoms ranging from dizziness, headache, and altered mental status. Loss of smell or taste has been associated with CNS involvement [2,3]. Clinically, about one-fourth of patients manifest CNS symptoms in the acute phase [1].

According to the International League Against Epilepsy and the International Bureau for Epilepsy, epilepsy is a brain disease characterized by an enduring predisposition to generate seizures and the neurobiological, cognitive, psychological, and social consequences of seizure recurrences [4]. It comprises an essential fraction of the worldwide disease burden, accounting for about 46 million people, 80% of whom are from low-income countries [5]. The effect of this pandemic in people with epilepsy (PWE) is relevant.

How and for how long SARS-COV-2 CNS infection affects the brain is an open debate [6]. It is too early to determine if remote symptomatic seizures will increase in patients with a history of COVID-19 or if non-provoked seizures will increase after the pandemic.

This narrative review discusses the potential pathophysiological mechanisms of COVID-19 on the brain, particularly in terms of direct CNS invasion, inflammatory response, and autoimmune responses. We discuss how CNS involvement may occur, its possible long-term consequences, and the impact the pandemic has had specifically in PWE.

Acute SARS-COV-2 related encephalopathy/encephalitis

Our current understanding of COVID-19 CNS involvement includes thromboembolic events resulting in hypoxia [7], blood-brain barrier alterations, and cerebral damage secondary to neuro-invasion and neuroinflammation [8]. Cytokine storm is a well-known pathophysiological mechanism of COVID-19, more common in adults over 65. When cytokines cross the brain-blood barrier, the astrocytes and microglia elicit a neuroinflammatory response, increasing oxidative stress and glutamate levels, leading to an excitotoxic environment with neuronal loss and parenchymal damage [9].

Among patients with COVID-19, 7.5 to 31% have presented with acute encephalopathy, manifesting altered mental status (i.e., amnesia, disorientation, behavioral changes, or sleep disorders) without evidence of encephalitis nor abnormalities on neuroimaging [3,10]. Most of these cases manifest a viral prodrome from 1 to 21 days [11].

A positive SARS-COV-2 genome sequencing or high titers of IgM antibodies against SARS- COV-2 spike protein in cerebrospinal fluid (CSF) samples have supported the diagnosis of COVID-19 encephalitis [11-14]. However, most cases of COVID-19-related encephalitis have shown no evidence of SARS- COV-2 in CSF [15]; polymerase chain reaction (PCR) analysis in CSF of 238 severe COVID-19 patients with neurological symptoms was positive for SARS-CoV-2 in only 6% [16]. Antibody testing in CSF is an alternative to making this diagnosis [17]. Besides, cases cataloged as COVID-19-related encephalitis with negative results for both tests have been reported [10]. Other authors have made the diagnosis of COVID-19-related encephalitis with no CSF testing [11,14]. The heterogeneity in these approaches makes interpretation challenging.

Individuals with COVID-19 encephalitis have shown poor responses to antiviral agents (i.e., remdesivir); however, a favorable response to rituximab may suggest an autoimmune setting [14,18]. A parallel with other well-known viral CNS infections associated with para-infectious autoimmune disorders might help to elucidate COVID-19 encephalitis pathophysiology. For instance, herpetic encephalitis has been related to anti-NMDAR encephalitis [19]. A comparison between herpetic encephalitis, anti-NMDAR-encephalitis, and COVID-19-related encephalitis is shown in Table 1. There is evidence of intrathecal pro-inflammatory cytokine production [12], mainly interleukin (IL)-6 and IL-8, in COVID-19 encephalitis [20]. Similarly, tumor necrosis factor (TNF)-alpha, IL-6, interferon (IFN)-alpha, IL-10, chemokine ligand (CXCL)13, and CXCL10 are all sensitive CSF markers of enteroviral encephalitis and anti-NMDAR encephalitis, corroborating CNS inflammation [21]. Proteinorrachia is common in patients with COVID-19 encephalitis, with occasionally lymphocytic pleocytosis [22], but is rarely seen in anti-NMDAR encephalitis.

**Table 1:** Clinical features of patients affected by COVID-19-related encephalitis, Herpetic encephalitis, or anti-NMDAR encephalitis. References: Garg et al., 2021 [10]; Tandon et al., 2021 [22]; Barry et al., 2015 [23]; Sili et al., 2014 [24]

	COVID-19 encephalitis	Herpetic encephalitis	Anti-NMDAR Autoimmune encephalitis
Age at presentation	> 50-years-old	> 50-years-old	Adolescent/young adults
Associated comorbidities	Hypertension, diabetes, or obesity	No specific associations	Ovarian teratoma (>50%)
Altered mental status	Common	Common	Common
Neuro-psychiatric features	Less common	Frequent behavioral changes	Prominent behavioral/ personality changes, psychosis
Cognitive changes	Long term, but not acutely	At presentation or long term	Prominent. At presentation or long term
Prodrome	Viral-like prodrome	Simultaneous	Viral-like prodrome
Other neurological symptoms	Movement disorders, autonomic manifestations	Headache Speech disturbances	Movement disorders, speech disturbances, autonomic manifestations
Seizures	Less likely	Frequent	Frequent
Laboratory findings	Lymphopenia Low platelets High urea levels	Leukocytosis Neutrophilia Hyponatremia	
Findings on EEG	Non-specific slowing in 90 to 100% of patients	Uni-or-bilateral periodic sharp waves or attenuation of amplitude, focal, generalized slow waves or epileptiform discharges.	Beta:Delta ratio (BDR), may be specific Extreme Delta brush (30% sensitivity) Unspecific slowing in 90% Continuous rhythmic activity during the catatonic phase
Findings on Brain MRI	Infrequent hyperintensities in different regions	Frequent temporal lobe involvement	Infrequent hyperintensities in different regions
Findings on CSF	Proteinorrachia Lymphocitic pleocytosis Inflammatory cytokines found on CSF	Proteinorrachia Lymphocitic pleocytosis Hypoglycorrhachia (25%) Inflammatory cytokines found on CSF	unfrequently proteinorrachia and Lymphocytic pleocytosis Oligoclonal bands (36%)
Mortality	22%	15-30%	5 to 10%
Long term Prognosis	Unknown	69% sequelae	75% recover or have mild sequelae

The most frequent findings in brain autopsies of people with severe COVID-19 were ischemic changes and reactive Alzheimer type II astrocytes. However, no specific results suggestive of meningitis or encephalitis were reported. Only five out of 18 patients had a positive SARS-CoV-2 PCR in this study [25].

Although infrequent, SARS-CoV-2-associated MRI changes can affect any part of the brain, with preferent temporal lobe involvement [11]. Leukoencephalopathy, acute necrotizing encephalitis, mild encephalitis/encephalopathy with a reversible splenial lesion (MERS), or posterior reversible encephalopathy syndrome (PRES) have been reported [3,26].

## Review

Short and long-term effects of COVID-19 in relation to epilepsy

1. Electroencephalogram (EEG) Features During the Acute Phase

1.1 Non-epileptiform EEG findings in patients with acute COVID-19: Acute EEG findings of critically ill patients with a COVID-19 diagnosis are non-specific [27]. For instance, a retrospective series of 19 patients undergoing continuous scalp EEG described non-convulsive seizures in 10.5% of patients and epileptiform discharges (including the two patients with seizures) in 15.7% of patients. Patients with altered mental status showed generalized polymorphic delta slowing in over 80% of their records. Absent background was seen in 42.1% of patients, slow background in 47.4% of patients, and a normal background was noted in 10.5% of all patients [28].

Over 1/3 of patients in a cohort of 22 had generalized periodic discharges (GPDs), primarily with triphasic morphology [28]. A systematic review noted that only 3.4% of 117 patients with COVID-19 connected to continuous scalp EEG due to altered mental status or seizure-like activity had a normal EEG. Over 60% of patients presented with generalized slowing and less than 8% with focal slowing [27]. Only 7,3% of patients had GPDs or lateralized periodic discharges in this review. Bilateral independent periodic discharges (BiPDs) were seen in only one patient, evolving into status epilepticus (SE) [27].

Among 36 in-hospital patients undergoing COVID-19 [27], 40 non-continuous scalp EEGs were performed due to delayed awakening after discontinued sedation or confusion. Almost 60% had either normal or mildly altered EEGs. No specific pathognomonic feature was noted [29].

As with previous studies, 19 out of 1574 patients with laboratory-confirmed COVID-19 required a scalp EEG [28]. Severe EEG abnormalities consistent with encephalopathy were seen in 13 of them. However, no periodic discharges or subclinical seizures were noted. Remarkably, five patients with severe encephalopathy showed an alpha coma pattern not attributed to sedation but related to the peak severity of COVID-19, between days 14 and 27 of disease. No difference in outcome was seen compared to those with severe encephalopathy and different EEG patterns [30].

Since alpha coma is generally a transient acute phenomenon, its late occurrence in patients with COVID-19 and normal neuroimaging might be secondary to neurotropism of COVID-19 and delayed CNS invasion [31].

A comparative analysis of quantitative EEG revealed significant differences in bandwidths between COVID-19 encephalopathy and other disorders. Patients with toxic or infectious encephalopathy had mainly delta frequencies within all lobes; in contrast, patients with post anoxic injuries due to cardiorespiratory arrest were seen to have higher rates of beta frequencies. COVID-19 was always in the middle ground for all bandwidth frequencies compared to these other pathologies [32]. Therefore, COVID-19 encephalopathy should not be considered only infectious nor strictly vascular.

A prospective Chilean study demonstrated that "requiring an EEG at the third week of COVID- 19 infection" was an independent risk factor for elevated mortality. This was possibly related to the hyperinflammatory phase, cytokine storm, and acute respiratory distress syndrome [33].

1.2 Interictal epileptiform discharges and non-convulsive SE in COVID-19 patients as compared to other encephalopathies: Epileptiform discharges are not frequent in patients with COVID-19 presenting with altered mental status [27]. Only 34 out of 177 COVID-19 patients monitored with EEG had interictal discharges (19.2%) in a systematic review, of whom two had a prior history of epilepsy. SE was noted in 4.5% of patients, and only five cases (2.8% of patients) had a non-convulsive status epilepticus (NCSE) [27]. Similarly, NCSE prevalence was 4.2% in a cohort of 62 patients suffering from severe COVID-19 [31]. The most frequent EEG finding was generalized continuous slowing. Although interictal activity was seen in almost 20% out of 94 EEGs analyzed from these 62 patients; the prevalence of SE was not different from other series [33].

A recent meta-analysis found EEG epileptiform discharges in 22.4% of patients with no history of epilepsy or seizures and up to 59.5% in those who had a history of epilepsy. Seizures were seen in no more than 2% of patients, and SE was seen in less than 1% of all patients [34]. Compared with herpetic encephalitis, with an incidence of close to 30%, the frequency of NCSE is much lower in COVID-19 [35]. The prevalence of SE in severe COVID-19 cases has been estimated to be as low as 5% [36].

In a recent multicenter retrospective study [37] including 4100 patients hospitalized with COVID-19, only 100 patients (2.68%) required an EEG during admission. As expected, most patients that required EEG needed mechanical ventilation. Almost one-fourth of these patients had a new-onset seizure, including 7% who presented with SE. Less than 15% of patients with seizures had a history of epilepsy [37]. A recent systematic review found that 27 out of 47 (57.4%) patients presented with SE after COVID-19 respiratory/gastrointestinal symptoms, with fewer patients (14.9%) developing SE as the first manifestation. Seventeen patients (36.2%) met the diagnostic criteria for New-onset Refractory SE (NORSE) [38].

2. Acute symptomatic seizures and COVID-19

Symptomatic seizures are uncommon during acute COVID-19 in adults, with a 0.5-1% incidence [1]. A retrospective study of 1043 patients reported a 0.7% seizure incidence, about 50% of whom have a history of epilepsy [39]. Among 22 patients with no history of epilepsy, only two had acute symptomatic focal seizures [28]. Both males were over 70 years old, had cardiovascular comorbidities, a history of cancer and severe COVID-19. None of them had brain imaging abnormalities.

In a cohort of 439 patients with confirmed or probable COVID-19, 4.3% presented with seizures [40]. Two of them had a history of epilepsy, and 14 had acute symptomatic seizures debatably related to COVID-19 infection. Five had a post-COVID-19 stroke, six had COVID-19-related encephalitis, and two had a stroke before COVID-19-infection. Only three had acute symptomatic seizures purely associated with COVID-19, with no prior history of epilepsy. These three patients did not share demographic characteristics with the previously mentioned studies. Age, sex, and phenomenology of seizures were not disclosed in this study.

Unlike in adults, seizures, including SE, can be the initial manifestation of COVID-19 in children, even in the absence of fever in children with no history of epilepsy. Seizures do not seem to be associated with disease severity [41].

3. Long term outcomes of COVID-19

3.1 Neurological post-acute sequelae of SARS-CoV-2 infection: Neurological post-acute sequelae of SARS-CoV-2 infection (PASC) comprise unspecific symptoms (smell/taste disturbance, neuromuscular manifestations, headache, and sleep disorders); neurocognitive (difficulty thinking/processing, short term memory loss, difficulty to focus); psychiatric symptoms (depression and anxiety, psychotic disorder and post-traumatic stress disorder), neuroendocrine manifestations and autonomic dysfunction [2].

A meta-analysis found that fatigue was the most frequently reported neurological manifestation of PASC, seen in 37% of patients, brain fog in 32%, sleep disturbances in over 1/3, and memory issues in 27% [42]. Patients with a less severe acute phase of infection had a higher prevalence of anxiety and depression. However, the lack of symptoms was associated with better scores on quality of life, and lower likelihood of depression, when compared to patients with symptoms [43]. On the other hand, memory complaints were seen more frequently in severe COVID-19 cases.

Although epilepsy does not seem to be a long-term effect of COVID-19 [6], patients with a prior diagnosis of epilepsy have been significantly affected. In a recent survey, among 153 patients with epilepsy depression rose from 19.7% at the beginning of the pandemic to 29.2%. Anxiety and insomnia did not increase significantly but remained high, and 17 patients (11.1%) presented increased seizure frequency [44].

3.2 Pathophysiology in PASC and the effects of vaccination: The pathophysiology of Neuro-PASC, and its long-term prognosis is not fully elucidated. In a survey conducted by Survivor Corps, about 40% of participants reported partial to complete resolution after vaccination. About 23% of Neuro-PASC patients in a small UK study were found to have no worsening in quality of life after vaccination. Moreover, 44 newly vaccinated individuals had a slight improvement. Symptom resolution occurs in 23.2% of vaccinated patients vs. 15.4% of unvaccinated patients [45].

A vaccine may act as a booster, improving an incomplete immune response in those with a virus reservoir, like in herpetic encephalitis; or like in post-viral encephalitis, CNS-related autoimmune disorders could arise post SARS-COV-2 infection [45] via "molecular mimicry", as reported in SARS-COV-1 and MERS-COV infections. 

As described with other viruses, a neuropathogenic mechanism of cognitive impairment might be due to chronic brain infection and release of viral proteins that harm nearby neurons and other cells [46]. An indirect route could be possible through macrophage proliferation, microglial/ astroglial activation, and dysregulated cytokine expression and production, resulting in synaptodendritic dysfunction, as described in HIV-associated neurocognitive disorders [47].

Higher immune and inflammatory set points with greater circulating biomarkers of inflammation are observed in mood disorders in the absence of known triggering factors. They are currently investigated as underpinning pathogenetic mechanisms for depressive psychopathology [48,49]. Peripheral cytokines involved in the host anti-viral response may elicit psychiatric and neurocognitive symptoms by precipitating inflammation in the peripheral and CNS. Inflammation is known to be associated with depression inducing blood-brain barrier (BBB) disruption, microglia activation, neurotransmission alteration, indoleamine 2,3-dioxygenase-1 activation, and oxidative stress [50,51].

Many studies have implied that inflammation activation is inextricably linked to cognitive dysfunction suggesting a primary role of IL-1β, IL-6, IL-18, and TNF-α Recently, elevated NLR was described in mild cognitive impairment, while platelet/lymphocyte ratio and SII were associated with the risk of dementia in the general population [52]. Unfortunately, targeted and long-term studies to test these hypotheses are warranted. It is necessary to standardize proper neurocognitive batteries to evaluate these patients over time and separate the cognitive evaluation from psychological or psychiatric symptoms.

3.3 “Brain-Fog”, and how people with epilepsy (PWE) have been affected by COVID-19: Current knowledge suggests that viral infections can trigger chronic inflammation and aberrant immune responses, causing long-lasting neuropsychiatric syndromes involving cognitive, affective, and behavioral symptoms from weeks to years following acute infection [51]. People with chronic illnesses are most at risk [53].

PWE may have diverse factors contributing to cognitive and behavioral impairments compared to the general population. These include elements related to the etiology or neuropathology of their epilepsy, history of SE, psychosocial factors such as education opportunities, and antiseizure medication (ASM) [54]. In that respect, ASM has known interactions with other treatments. Treatment with ASM that interact with cytochrome p450 enzymes may generate hepatic problems, which may become exacerbated by severe COVID-19 infection. Similarly, patients with severe COVID-19 disorders may have multi-organ failure that could require adjustment or even change in their ASM [55].

Recently, long-term neurological symptoms of COVID-19 have been divided according to their temporality: (1) subacute, from 4 to 12 weeks beyond the acute phase of COVID-19, and (2) chronic or post COVID-19 syndrome after 12 weeks [56]. The most common abnormalities seen in the latter include fatigue, myalgia, headache, dysautonomia, and cognitive impairment (or brain fog); mimicking fibromyalgia and chronic fatigue syndrome [2].

In a prospective study of 1733 patients in Wuhan, at six months of COVID-19 infection, ~80% reported at least one post-COVID-19 symptom. Fatigue or muscle weakness was reported in 63%, sleep difficulties in 26%, and anxiety or depression in 23% [57].

Although seizures do not seem to be an essential acute complication related to COVID-19 [35,38], people with known epilepsy have seen an increase in seizure frequency since the start of the pandemic [58]. A survey by the ILAE task force showed that this increase in seizure frequency might be multifactorial and includes difficulties accessing healthcare systems, accessing information, difficulty accessing psychological support, and an increase in psychological distress. Barriers to access to the usual health programs included delayed investigation and therapy, appointment cancellations, limited availability of providers, and dissatisfaction with tele-health. Regarding the increase in psychological stress, this same study found that 57.1% of PWE had a K-6 score higher than 13, compared to 14% in a similar cohort surveyed before the pandemic [58].

It is essential to study the effect epilepsy and ASMs may have on long-haul COVID-19. Brain fog is a significant long-term effect of COVID-19 infection. Patients with epilepsy may be using ASM, which could have crucial cognitive side effects. Newer ASMs such as lamotrigine, levetiracetam, and gabapentin have been noted to have fewer cognitive effects when compared to carbamazepine [59]. On the other hand, topiramate has a poor cognitive profile, with an essential impact on verbal fluency [59]. Although levetiracetam seems to be safe from a metabolic and cognitive perspective, its effect from a neuropsychological standpoint could influence the long-term psychiatric disorders noted as part of long haul covid, such as anxiety and depression.

## Conclusions

In contrast with other CNS infections, patients with COVID-19 have a lower incidence of acute symptomatic seizures and SE, but long-term neurological symptoms have been noted. Neuropsychiatric symptoms such as depression and neurocognitive impairment have been observed in patients with COVID-19 months after acute infection. Brain fog, confusion, or mild cognitive complaints in the convalescent phase after acute COVID-19 encephalitis suggests chronic encephalopathy. PWE present with distinct elements that may exacerbate cognitive and behavioral impairments compared to the general population. These include but are not limited to the effect of antiseizure medication (ASM) during or after COVID-19 infection.

In patients over 65 years of age with a history of mild cognitive impairment or dementia, SARS-CoV-2 could accelerate underlying neurodegeneration, provoking an exaggerated decline in neuropsychological functions. Although rare, acute symptomatic seizures have also been reported. So far, the incidence of epilepsy after COVID-19 infection is yet to be determined. 

Available information on seizure incidence in patients with COVID-19 is scarce. Patient outcomes of those manifesting seizures or SE during acute illness are heterogeneous. Longer follow-up is needed to elucidate the prognosis of acute seizures and SE related to COVID-19 and the risk of developing epilepsy. Regarding patients with prior known epilepsy, the pandemic seems to have had significant effects on quality of life and seizure frequency. 

Long-term neurological symptoms linger in patients with a history of COVID-19, with neuropsychiatric symptoms persisting even after three months of infection. Future investigations should focus on determining seizure incidence in patients with COVID-19 and longer follow-up is needed to elucidate the prognosis of acute seizures and SE related to COVID-19 and the risk of developing epilepsy. How COVID-19 may affect patients' long-term quality of life is still an open question. Standardized cognitive batteries are needed to make a reliable and homogeneous neurocognitive assessment and in the laboratory setting would be interesting to assess immunological pathways involved through the identification of different innate and adaptive immunity biomarkers.
